# A 1-year analysis of adverse events following COVID-19 vaccination in Lebanon: a retrospective study

**DOI:** 10.1186/s40545-023-00528-1

**Published:** 2023-02-21

**Authors:** Abeer Zeitoun, Souheil Hallit, Sirine Chehade, Aya Ibrahim, Maya Helali, Carla Allam, Rita Karam

**Affiliations:** 1 Quality Assurance of Pharmaceutical Products Department, National Pharmacovigilance Program, Lebanese Ministry of Public Health, Beirut, Lebanon; 2 Faculty of Medicine and Medical Sciences, Holy Spirit University of Kaslik, Jounieh, Lebanon; 3 Department of Chemistry and Biochemistry, Faculty of Sciences, Lebanese University, Beirut, Lebanon; 4 Pharmacology Department, Faculty of Medical Sciences, Lebanese University, Beirut, Lebanon

**Keywords:** Pharmacovigilance, COVID-19 vaccines, Adverse event following immunization, SARS-CoV-2

## Abstract

**Background:**

Since the deployment of Coronavirus Disease 2019 (COVID-19) vaccines, skepticism about the safety, incidence, and severity of Adverse Events Following Immunization (AEFI) was a concern. The study has two main objectives. First, to analyze AEFIs following COVID-19 vaccines (Pfizer-BioNTech, AstraZeneca, Sputnik, and Sinopharm) during the vaccination campaign in Lebanon and correlate them with age and gender. Second, to correlate Pfizer-BioNTech and AstraZeneca vaccines’ AEFI with the dose administered.

**Methods:**

A retrospective study was carried out between February 14th, 2021, and February 14th, 2022. AEFI case reports received to the Lebanese Pharmacovigilance (PV) Program were cleaned, validated, and analyzed using SPSS software.

**Results:**

A total of 6808 AEFI case reports were received to the Lebanese PV Program during the period of this study. Case reports were mostly received from females (60.7%) and from vaccine recipients aged 18–44 years. As for the vaccine type, AEFIs occurred more frequently with the AstraZeneca vaccine compared to the Pfizer-BioNTech vaccine. The latter had AEFIs mainly following dose 2, whereas AEFIs with the AstraZeneca vaccine were more frequently reported after dose 1, with general body pain being the most reported systemic AEFI with PZ (34.6%), while fatigue was the most reported AEFI with AZ vaccine (56.5%).

**Conclusions:**

The AEFI reported with COVID-19 vaccines in Lebanon were aligned with those reported worldwide. The incidence of rare serious AEFIs should not discourage the public from getting vaccinated. Further studies are needed to evaluate their long-term potential risk.

## Introduction

In December 2019, a novel coronavirus was identified in Wuhan, China, following a cluster of pneumonia cases. Shortly after, in January 2020, WHO officials confirmed a case of Corona Virus Disease 2019 (COVID-19) in Thailand; the first case that was recorded outside of China. On January 30, 2020, the WHO declared the novel coronavirus outbreak a Public Health Emergency of International Concern (PHEIC). Alarmed by the levels of spread and seriousness of this virus, COVID-19 was declared a pandemic by the WHO on March 11th, 2020 [1]. Until February 2022, over 420 million cases of COVID-19 infection have been confirmed and over 5.8 million cases of mortality have been reported internationally [2].

Before the development of COVID-19 vaccines, several precautions were put in place to help control the spread. These included physical distancing, wearing facemasks, travel restrictions, and lockdowns [3]. Nevertheless, the need for vaccines against this pandemic seemed to be the most efficient way to overcome this global crisis [4]. Following the publication of the genetic sequence of COVID-19 disease also referred to as Severe Acute Respiratory Syndrome Coronavirus 2 (SARS-CoV-2) on 11 January 2020, the race to develop vaccines against this disease was initiated. A diversity of technology platforms was evaluated to develop different types of COVID-19 vaccines including mRNA (Pfizer-BioNTech and Moderna), adenoviral (AstraZeneca and Sputnik), inactivated virus (Sinovac and Sinopharm), and protein subunits vaccines (Novavax) [5–7]. Several studies have been published reporting high vaccine effectiveness. A study conducted in England showed that getting vaccinated with either one dose of Pfizer-BioNTech (PZ) or AstraZeneca (AZ) was associated with a similar effect of significant reduction in symptoms related to COVID-19 and protection against severe diseases in older adults [8].

In Lebanon, the first COVID-19 positive case was reported on the 21st of February 2020 [9]. Consequently, the Ministry of Public Health imposed strict measures in accordance with WHO recommendations. The number of infected individuals started rising and on the 10th of March 2020 the first case of COVID-19-related death was announced. Until February 2022, the 6.9-million population country had over 1,097,118 infections (182,853 cases per million) and 10,392 coronavirus-related deaths (1732 per million) reported since the beginning of the pandemic [10].

On December 14, 2020, mass vaccination campaigns started in the Unites States; shortly after, they were launched across European countries [11, 12]. The first COVID-19 vaccine that reached Lebanon was the PZ vaccine after it has been granted an Emergency Use Authorization (EUA) in December 2020. A nationwide COVID-19 vaccination campaign was established by the Lebanese Ministry of Public Health (MoPH) on February 14th, 2021. Following the introduction of the PZ vaccination campaign, immunization with AZ, Sputnik, and Sinopharm COVID-19 vaccines was initiated [13]. In Lebanon, 5,134,400 doses of COVID-19 vaccines have been administered up to the date of this current study. Consequently, 40.9% of the country’s population have been fully immunized with primary vaccine series, i.e., received dose 1 and 2 of their vaccine [10].

Similar to any immunization process, COVID-19 vaccines may be associated by adverse events, also called Adverse Event Following Immunization (AEFI). According to the WHO, an AEFI is any “untoward medical occurrence following vaccination that may be any unfavorable or unintended sign, abnormal laboratory finding, symptom or disease” [14]. As for the AZ and PZ vaccines, they were shown to have acceptable short-term safety outcomes, similar duration, severity, and incidence of adverse events in comparison with clinical trials [15]. Common AEFI with COVID-19 vaccines include injection site reactions, fever, tiredness, headache, chills, and muscle pain [16]. Studies have described the occurrence of severe adverse reactions temporally associated with COVID-19 vaccines, such as Bell’s palsy, myocarditis, deep vein thrombosis, and anaphylactic reactions [17–20] .

Skepticism regarding the safety of the vaccine was driven by many factors, such as the accelerated development of vaccines, fear of long-term adverse events, and lack of knowledge about the benefit to risk ratio of immunization. Vaccine hesitancy and refusal was fueled by news spreading throughout the media and misinformation was hard to dislodge. Consequently, concerns were raised, and anxiety was spread among the public [3, 21].

This study includes data from February 14th, 2021, and February 14th, 2022. The objective of this 1-year analysis is to describe AEFIs following COVID-19 vaccines (PZ, AZ, Sputnik, and Sinopharm) reported during the national vaccination campaign through passive surveillance system and correlate them with age and gender. Since PZ and AZ vaccines were mainly administered in Lebanon, correlation between these vaccines and dose number administered was also performed. The aim of the latter is to prompt policymakers to develop recommendations and guidelines.

## Methods

### AEFI reporting system

Within the scope of the AEFIs surveillance related to the available COVID-19 Vaccines in Lebanon, the Pharmacovigilance (PV) Program established a procedure for the management of reported AEFIs.

AEFIs can be reported through one of the following means:

- The COVID-19 Vaccine Hotline Call Center, 1214, created by the MoPH [22],
- The IMPACT Platform which stands for the Inter-Ministerial and Municipal Platform for Assessment, Coordination and Tracking. It is the first e-Governance platform in Lebanon that includes data about COVID-19 Coordination. Individuals wishing to receive their COVID-19 vaccination should register on this platform. In addition, the latter can be used to report any AEFI [23, 24],
- The Kobo toolbox, a Software created by the MoPH for vaccination centers to report AEFIs [25],
- Direct contact with the PV Program from other departments in the MoPH.

The data collected through these means include the name, contact details, age, and gender of the reporter, the vaccine administered, and the adverse event(s) temporally associated with the vaccine in question.

A case report refers to a report received by the PV Program, which pertains to one individual vaccine recipient who reported at least one AEFI with COVID-19 vaccines.

### Data analysis

The retrospective study included AEFIs received through passive surveillance over 1 year. All case reports were screened and validated for data completion. Incomplete or inconsistent case reports were followed-up directly with the initial reporter. Case reports were classified as serious or non-serious. In this study, a case report is classified as serious if it contains at least one serious AEFI as per the World Health Organization (WHO) definition. According to the WHO, an AEFI is defined as ‘serious’ if it meets one or more of the following criteria: results in death; is life-threatening; requires inpatient hospitalization or prolongation of an existing hospitalization; results in persistent or significant disability/incapacity; is a congenital anomaly/birth defect; and/or is a medically important event or reaction [26] .

The non-serious case reports were directly entered into the national web-based Individual Case Safety Report (ICSR) data management system, VigiFlow, which supports the collection, processing, analyzing and sharing of Adverse Drug Reaction (ADR) and AEFI reports [27, 28]. The data entry into this system was done by coding the description provided by the reporter into standardized terms using the Medical Dictionary for Regulatory Activities (MedDRA^®^). As for the serious case reports, the PV team follow-up with the reporter and his physician, investigate the case using the WHO AEFI investigation form [29], and conducts a causality assessment using the WHO AEFI Causality Assessment Software [30]. Subsequently, the Serious AEFI Special Committee assess the cases with a final decision before their entry into VigiFlow. This committee was appointed in April 2021 through a ministerial decision (603/1) by the MoPH to evaluate serious AEFIs with COVID-19 vaccine [31].

### Data extraction

The adverse events assessed in this study covered a 1-year period from February 14th, 2021, to February 14th, 2022. Case reports were extracted after data cleaning and validation on February 14th, 2022, from VigiLyze which is a World Health Organization–Uppsala Monitoring Centre (WHO–UMC) signal detection and signal management tool [32, 33]. The number of vaccine doses per Lebanese governorate during this period was obtained from the MoPH official website [34].

### Statistical analysis

Statistical analyses were performed using SPSS software (version 23.0). Categorical variables were compared using Pearson’s *χ*^2^ test. Continuous variables were compared using the Mann–Whitney *U* and Kruskal–Wallis tests. Statistical significance was set at *p* < 0.05.

## Results

### Description of doses administered and case reports following COVID-19 vaccines

Following the administration of 5,134,093 doses of COVID-19 vaccines in Lebanon between February 14th, 2021, and February 14th, 2022, a total of 6808 case reports were received to the PV Program which translates into a reporting rate of 1.33 case reports per 1000 doses administered, 0.99 per 1000 doses administered (4255 case reports) for the PZ vaccine and 3.2 per 1000 doses (2302 case reports) for the AZ vaccine.

Case reports were mostly received from females (60.7%) and from vaccine recipients aged between 18 and 44 years (55.33%). Out of the case reports received, 76.84% were after the 1st dose (Table 1).

**Table 1:** Description of doses administered and case reports following COVID-19 vaccines

Characteristics	*N* (%)
Administered doses	5,134,093
Vaccine type	
Pfizer-BioNTech	4,259,387 (82.96)
AstraZeneca	717,370 (13.97)
Sputnik V	123,520 (2.41)
Sinopharm	17,934 (0.35)
Case reports	6808
Seriousness criteria	
Serious case reports	452 (6.64)
Non-serious case reports	6356 (93.36)
Non-serious case reports by vaccine type	
Pfizer-BioNTech	3955 (62.20)
AstraZeneca	2164 (34.10)
Sputnik V	225 (3.50)
Sinopharm	12 (0.20)
Gender	
Female	4135 (60.7)
Male	2673 (39.3)
Age	
12–17	248 (3.67)
18–44	3741 (55.33)
45–64	1999 (29.57)
65–74	350 (5.18)
> 75	423 (6.25)
Onset of AEFIs	
After dose 1	5231 (76.9)
After dose 2	1369 (20.12)
After dose 3	202 (2.98)
Governorates	6642 (100)
Mount Lebanon^a^	2845 (42.83)
Beirut^b^	2015 (30.34)
North Lebanon^c^	852 (12.83)
South Lebanon^d^	597 (9.0)
Bekaa/Baalbeck-Hermel^e^	333 (5.0)
Means of reporting	
IMPACT platform	3829 (56.3)
1214 Hotline Call Center	1979 (29.0)
Vaccination centers	924 (13.6)
Others	76 (1.1)

Results are presented as frequency (*N*) and percentage (%)

*AEFI* Adverse Event Following Immunization

^a^Mount Lebanon governorate includes vaccination centers in Aley, Baabda, Chouf, Matn, Jbeil, Keserwan, and Baskinta

^b^Beirut governorate includes vaccination centers in Beirut area

^c^North Lebanon governorate includes vaccination centers in Batroun, Bcharreh, Koura, Minieh-Danniyeh, Tripoli, and Akkar

^d^South Lebanon governorate includes vaccination centers in Jezzine, Saida, Tyre, Bint Jbeil, Hasbaya, and Marjeyoun

^e^Bekaa/Baalbeck-Hermel governorate includes vaccination centers in Rashaya, West Bekaa, Zahleh, Baalbeck, and Hermel

### Reported COVID-19 vaccine adverse events and their correlation with age and gender

General body pain (41.8%), headache (38.5%), chills (31.2%), nausea (19.7%), abdominal pain (8.4%), vomiting (6.4%), injection site pain (43.9%), injection site swelling (11.0%) and injection site erythema (7.7%) were more significant in females than in males (*p* < 0.05). The incidence of the remaining AEFIs was higher in females than in males; however, results were not found to be statistically significant (*p* > 0.05). The only AEFI that was reported more often in males was fever (33.5%).

Vaccine recipients aging between 18 and 44 years had a statistically significant reporting percentage in comparison with other age groups in the majority of AEFIs (*p* < 0.05) (Table 2).

**Table 2:** Reported COVID-19 vaccine adverse events and their correlation with age and gender

AEFI	Gender			Age group (years)					
	Female *N* = 3847 (%)	Male *N* = 2509 (%)	*p* value	12–17 *N* = 233 (%)	18–44 *N* = 3546 (%)	45–64 *N* = 1864 (%)	65–74 *N* = 312 (%)	> 75 *N* = 372 (%)	*p* value
Systemic AEFIs									
Fatigue	1603 (41.7)	1009 (40.2)	0.251	57 (24.5)	1579 (44.5)	769 (41.3)	114 (36.5)	92 (24.7)	< 0.001*
General body pain	1609 (41.8)	969 (38.6)	0.011*	38 (16.3)	1532 (43.2)	761 (40.8)	107 (34.3)	131 (35.2)	< 0.001*
Headache	1482 (38.5)	859 (34.2)	0.001*	58 (24.9)	1452 (40.9)	691 (37.1)	94 (30.1)	42 (11.3)	< 0.001*
Fever	1238 (32.2)	840 (33.5)	0.286	65 (27.9)	1,248 (35.2)	612 (32.8)	72 (23.1)	76 (20.4)	< 0.001*
Chills	1202 (31.2)	706 (28.1)	0.009*	31 (13.3)	1118 (31.5)	602 (32.3)	65 (20.8)	87 (23.4)	< 0.001*
Nausea	757 (19.7)	290 (11.6)	< 0.001*	29 (12.4)	677 (19.1)	281 (15.1)	38 (12.2)	21 (5.6)	< 0.001*
Dyspnea	308 (8.0)	198 (7.9)	0.887	17 (7.3)	349 (9.8)	116 (6.2)	12 (3.8)	12 (3.2)	< 0.001*
Abdominal pain	323 (8.4)	165 (6.6)	0.008*	19 (8.2)	338 (9.5)	108 (5.8)	13 (4.2)	10 (2.7)	< 0.001*
Diarrhea	287 (7.5)	168 (6.7)	0.253	14 (6.0)	279 (7.9)	118 (6.3)	25 (8.0)	19 (5.1)	0.102
Cough	260 (6.8)	159 (6.3)	0.535	13 (5.6)	287 (8.1)	92 (4.9)	14 (4.5)	13 (3.5)	< 0.001*
Dizziness	240 (6.2)	134 (5.3)	0.141	41 (17.6)	220 (6.2)	85 (4.6)	18 (5.8)	10 (2.7)	< 0.001*
Vomiting	247 (6.4)	94 (3.7)	< 0.001*	15 (6.4)	202 (5.7)	97 (5.2)	12 (3.8)	14 (3.8)	0.428
Local AEFIs									
Injection site pain	1687 (43.9)	1020 (40.7)	0.012*	63 (27.0)	1669 (47.1)	765 (41.0)	125 (40.1)	78 (21.0)	< 0.001*
Injection site swelling	424 (11.0)	188 (7.5)	< 0.001*	17 (7.3)	380 (10.7)	177 (9.5)	26 (8.3)	10 (2.7)	< 0.001*
Injection site erythema	296 (7.7)	113 (4.5)	< 0.001*	8 (3.4)	241 (6.8)	128 (6.9)	15 (4.8)	15 (4.0)	0.044*

Results are presented as frequency (*N*) and percentage (%)

*AEFI* Adverse Event Following Immunization

^*^p < 0.05

### Reported adverse events with PZ and AZ COVID-19 vaccine and their correlation with the dose number

Regarding the PZ vaccine, AEFIs were mainly received following dose 2. However, injection site erythema (6.9%) was more frequently reported after dose 1, whereas injection site swelling (12.2%) and dizziness (7.9%) were predominant after dose 3 (Table 3).

**Table 3:** Reported adverse events with PZ vaccine and their correlation with the dose number

PZ vaccine AEFIs	Dose 1 *N* = 2711 (%)	Dose 2 *N* = 1051 (%)	Dose 3 *N* = 189 (%)	*p* value
Systemic AEFIs				
General body pain	894 (33.0)	406 (38.6)	54 (28.6)	0.001*
Fatigue	700 (25.8)	483 (46.0)	53 (28.0)	< 0.001*
Headache	721 (26.6)	385 (36.6)	44 (23.3)	< 0.001*
Fever	562 (20.7)	352 (33.5)	59 (31.2)	< 0.001*
Chills	516 (19.0)	313 (29.8)	42 (22.2)	< 0.001*
Nausea	287 (10.6)	178 (16.9)	32 (16.9)	< 0.001*
Dyspnea	174 (6.4)	108 (10.3)	12 (6.3)	< 0.001*
Abdominal pain	135 (5.0)	104 (9.9)	7 (3.7)	< 0.001*
Diarrhea	165 (6.1)	70 (6.7)	12 (6.3)	0.807
Cough	151 (5.6)	83 (7.9)	13 (6.9)	0.028*
Dizziness	175 (6.5)	63 (6.0)	15 (7.9)	0.592
Vomiting	92 (3.4)	69 (6.6)	8 (4.2)	< 0.001*
Local AEFIs				
Injection site pain	898 (33.1)	490 (46.6)	64 (33.9)	< 0.001*
Injection site swelling	265 (9.8)	105 (10.0)	23 (12.2)	0.567
Injection site erythema	188 (6.9)	51 (4.9)	7 (3.7)	0.02*

Results are presented as frequency (*N*) and percentage (%)

*AEFI* Adverse Event Following Immunization

*p < 0.05

Table 4 shows that the majority of AEFIs with the AZ vaccine primarily occurred after the dose 1. As for dyspnea (9.1%) and cough (7.3%), their incidence was higher following the second dose. To note, two case reports which included general body pain, fatigue, headache, fever, and injection site pain as AEFIs were received after dose 3.

**Table 4:** Reported adverse events with AZ vaccine and their correlation with the dose number

AZ vaccine AEFIs	Dose 1 *N* = 1997 (%)	Dose 2 *N* = 164 (%)	*p* value
Systemic AEFIs			
General body pain	1047 (52.4)	54 (32.9)	< 0.001*
Fatigue	1152 (57.7)	68 (41.5)	< 0.001*
Headache	1026 (51.4)	51 (31.1)	< 0.001*
Fever	958 (48.0)	44 (26.8)	< 0.001*
Chills	887 (44.4)	39 (23.8)	< 0.001*
Nausea	470 (23.5)	25 (15.2)	0.015*
Dyspnea	180 (9.0)	15 (9.1)	0.954
Abdominal pain	217 (10.9)	10 (6.1)	0.056
Diarrhea	175 (8.8)	12 (7.3)	0.527
Cough	144 (7.2)	12 (7.3)	0.960
Dizziness	106 (5.3)	7 (4.3)	0.565
Vomiting	153 (7.7)	12 (7.3)	0.873
Local AEFIs			
Injection site pain	1078 (54.0)	56 (34.1)	< 0.001*
Injection site swelling	194 (9.7)	10 (6.1)	0.128
Injection site erythema	143 (7.2)	7 (4.3)	0.161

Results are presented as frequency (*N*) and percentage (%)

*AEFI* Adverse Event Following Immunization

*p < 0.05

### Comparison of reported adverse events with PZ and AZ vaccines and their correlation with the vaccine’s first, second, and both doses

The results revealed a significant increase (*p* < 0.05) in the incidence of most AEFIs after the first dose of the AZ vaccine in comparison with the PZ vaccine. Following dose 2, higher percentages of AEFIs were noticed with the PZ vaccine than with the AZ vaccine except for dizziness (PZ 6.7%, AZ 7.3%) and vomiting (PZ 6.6%, AZ 7.3%). Findings also revealed a rise in the number of AEFIs with both doses of the AZ vaccine compared to the PZ vaccine, except for dizziness (PZ 6.3%, AZ 5.2%) and injection site swelling (PZ 9.8%, AZ 9.4%) (Table 5).

**Table 5:** Comparison of reported adverse events with PZ and AZ vaccines and their correlation with the vaccine’s first, second, and both doses

AEFI	Dose 1			Dose 2			Dose 1 and 2		
	PZ *N* = 2711 (%)	AZ *N* = 1997 (%)	*p* value	PZ *N* = 1051 (%)	AZ *N* = 164 (%)	*p* value	PZ *N* = 3762 (%)	AZ *N* = 2161 (%)	*p* value
Systemic AEFIs									
General body pain	894 (33.0)	1047 (52.4)	< 0.001*	406 (38.6)	54 (32.9)	0.161	1300 (34.6)	1101 (50.9)	< 0.001*
Fatigue	700 (25.8)	1152 (57.7)	< 0.001*	483 (46.0)	68 (41.5)	0.282	1183 (31.4)	1220 (56.5)	< 0.001*
Headache	721 (26.6)	1026 (51.4)	< 0.001*	385 (36.6)	51 (31.1)	0.169	1106 (29.4)	1077 (49.8)	< 0.001*
Fever	562 (20.7)	958 (48.0)	< 0.001*	352 (33.5)	44 (26.8)	0.090	914 (24.3)	1002 (46.4)	< 0.001*
Chills	516 (19.0)	887 (44.4)	< 0.001*	313 (29.8)	39 (23.8)	0.115	829 (22.0)	926 (42.9)	< 0.001*
Nausea	287 (10.6)	470 (23.5)	< 0.001*	178 (16.9)	25 (15.2)	0.589	465 (12.4)	495 (22.9)	< 0.001*
Dyspnea	176 (6.4)	180 (9.0)	0.001*	108 (10.3)	15 (9.1)	0.656	282 (7.5)	195 (9.0)	0.038*
Abdominal Pain	135 (5.0)	217 (10.9)	< 0.001*	104 (9.9)	10 (6.1)	0.121	239 (6.4)	227 (10.5)	< 0.001*
Dizziness	165 (6.1)	175 (8.8)	< 0.001*	70 (6.7)	12 (7.3)	0.755	238 (6.3)	113 (5.2)	0.085
Diarrhea	151 (5.6)	144 (7.2)	0.022*	83 (7.9)	12 (7.3)	0.797	235 (6.2)	187 (8.7)	0.001*
Cough	175 (6.5)	106 (5.3)	0.101	63 (6.0)	7 (4.3)	0.378	234 (6.2)	156 (7.2)	0.136
Vomiting	92 (3.4)	153 (7.7)	< 0.001*	69 (6.6)	12 (7.3)	0.720	161 (4.3)	165 (7.6)	< 0.001*
Local AEFIs									
Injection site pain	898 (33.1)	1078 (54.0)	< 0.001*	490 (46.6)	56 (34.1)	0.003*	1388 (36.9)	1134 (52.5)	< 0.001*
Injection site swelling	265 (9.8)	194 (9.7)	0.945	105 (10.0)	10 (6.1)	0.113	370 (9.8)	204 (9.4)	0.621
Injection site redness	188 (6.9)	143 (7.2)	0.764	51 (4.9)	7 (4.3)	0.744	239 (6.4)	150 (6.9)	0.379

Results are presented as frequency (*N*) and percentage (%)

*AEFI* Adverse Event Following Immunization

*p < 0.05

### Serious cases following COVID-19 vaccines and their correlation with age, gender, dose number, and time to onset

Out of the 452 serious cases received, 68 were investigated and assessed for causality with a final decision by the Serious AEFI Special Committee. Out of the investigated cases, 55 (80.88%) were temporally associated with the PZ vaccine and 13 (19.12%) with the AZ vaccine.

The mean age of the 55 case reports was 66.9 ± 20.6 years, 30 were males (54.55%) and 25 were females (45.45%). There were 30 cases (54.55%) that occurred 7.6 ± 7.31 days following the first dose, 22 cases (40.0%) that occurred 11.33 ± 20.91 days following the second dose, and 3 cases (5.45%) that occurred 7.33 ± 11.85 days following the third dose. There were 15 cases of death (27.27%) and 40 cases of hospitalization (72.73%). The Serious AEFI Special Committee classified 5 serious cases as consistent (9.09%) with PZ vaccine. These include one case of Anaphylactic Shock, Myocarditis, Pericarditis, and two cases of Guillain–Barre Syndrome.

As for 13 serious cases temporally associated with AZ vaccine, the mean age group was 49.8 ± 11.3 years. There were 6 males (76.15%) and 7 females (23.85%). There were 10 cases (76.93%) that occurred 11.4 ± 9.5 days following the first dose, and 2 cases (15.38%) that occurred 3.5 ± 2.12 days following the second dose. There was one case (7.69%) of immunization error, where the patient received both doses 1 and 2 during the same vaccination session and had a serious AEFI (Myocardial Infarction) 9-day post-vaccination. Moreover, there were 4 cases of death (30.77%) and 9 cases of hospitalization (69.23%). The Serious AEFI Special Committee classified 3 serious cases as consistent (23.08%) with AZ vaccine. These include cases of Vaccine-Induced Thrombotic Thrombocytopenia, Cerebral Hemorrhage, and Myocardial Infarction (Table 6).

**Table 6:** Serious cases following COVID-19 vaccines and their correlation with age, gender, dose number, and time to onset

	Total	PZ	AZ	*p* value
Case report, *N* (%)	68 (100)	55 (80.88)	13 (19.12)	
Age, Mean ± SD	63.62 ± 20.23	66.9 ± 20.6	49.8 ± 11.3	0.002*
Gender, *N* (%)				
Male	36 (52.9)	30 (54.55)	6 (76.15)	0.586
Female	32 (47.1)	25 (45.45)	7 (53.85)	
Onset of AEFIs, *N* (%)				
After dose 1	40 (58.8)	30 (54.55)	10 (76.93)	0.089
After dose 2	24 (35.3)	22 (40)	2 (15.38)	
After dose 3	3 (4.4)	3 (5.45)	0	
After dose 1 and 2^a^	1 (1.5)	0	1 (7.69)	
TTO per dose administered, Mean ± SD				
After dose1	8.55 ± 7.96	7.6 ± 7.31	11.4 ± 9.5	0.706
After dose 2	10.63 ± 19.63	11.33 ± 20.91	3.5 ± 2.12	
After dose 3	7.33	7.33 ± 11.85	–	
After dose 1 and 2^a^	9	–	9	
Seriousness criteria, *N* (%)				
Fatal	19 (27.9)	15 (27.27)	4 (30.77)	0.801
Hospitalized	49 (72.1)	40 (72.73)	9 (69.23)	
AEFI Committee decision, *N* (%)				
Coincidental	36 (52.9)	31 (56.36)	5 (38.46)	0.25
Indeterminate	24 (35.3)	19 (34.55)	5 (38.46)	
Consistent	8 (11.8)	5 (9.09)	3 (23.08)	

Results are presented as frequency (*N*) and percentage (%) or as mean ± Standard Deviation (SD)

*AEFI* Adverse Event Following Immunization, *TTO* time to onset in days

^*^p < 0.05

^a^This is an immunization-error case in which the patient received both doses during the same vaccination session

### Reported serious cases by system organ class

Out of the 68 assessed serious cases, cardiovascular disorder (*N* = 46, 67.6%) was the most frequent MedDRA System Organ Class (SOC) associated with the occurrence of AEFIs following both PZ (*N* = 38, 69.1%) and AZ (*N* = 8, 61.5%) COVID-19 vaccines (Table 7).

**Table 7:** Reported serious cases by system organ class (SOC)

System organ class	Total *N* = 68 (100%)	PZ *N* = 55 (80.88%)	AZ *N* = 13 (19.12%)	*p* value
Cardiovascular disorders^a^	46 (67.6)	38 (69.1)	8 (61.5)	0.743
Nervous system disorders^b^	8 (11.7)	6 (10.9)	2 (15.4)	0.643
Infections and infestations^c^	7 (10.3)	6 (10.9)	1 (7.7)	1.0
Respiratory, thoracic, and mediastinal disorders^d^	2 (3.0)	2 (3.6)	0 (0.0)	1.0
Blood and lymphatic system disorders^f^	1 (1.5)	0 (0.0)	1 (7.7)	0.191
Surgical and medical procedures^g^	1 (1.5)	0 (0.0)	1 (7.7)	0.191
Immune system disorders^e^	3 (4.4)	3 (5.5)	0 (0.0)	1.0

Results are presented as frequency (*N*) and percentage (%)

System Organ Classes (SOC) are groupings by etiology (such as infections and infestations), manifestation site (such as gastrointestinal disorders) or purpose (such as surgical and medical procedures)

^a^Includes case reports of cardiac arrest, cerebrovascular accident, myocardial infarction, myocarditis, pericarditis, atrial fibrillation, extensive portal vein thrombosis, unstable angina, Kounis syndrome, and thrombotic disorders

^b^Includes case reports of Guillain–Barré syndrome, acute disseminated encephalomyelitis, amyotrophic lateral sclerosis, cerebral hemorrhage, and epilepsy

^c^Includes case reports of pneumonia, acute bronchitis, and sepsis

^d^Includes case reports of dyspnea, polypnea, and pulmonary edema

^e^Includes case reports of acute severe urticaria, anaphylactic shock, and hyperstimulation of the immune system

^f^Includes case reports febrile neutropenia and vaccine-induced immune thrombotic thrombocytopenia (VITT)

^g^Includes case reports of post-surgical bleeding

## Discussion

Vaccine reluctance represents a serious health threat worldwide. Like any other vaccines, hesitancy can hinder the successful control of the spread of the COVID-19 pandemic [35]. A study by Hanna et al. found that common barriers to receive COVID-19 vaccines in Lebanon included both the fear of potential long-term and short-term adverse events [36]. Thus, post-marketing surveillance of AEFIs is a fundamental part of any immunization program including COVID-19 vaccines.

Observations of the current study on the gender and AEFIs with all four COVID-19 vaccines (AZ, PZ, Sputnik, and Sinopharm) showed a higher reporting rate of AEFIs among females (61.7%) than males (39.3%). These results were in accordance with findings of the Saudi Arabian study by El-Shitany et al. which revealed a higher reporting of AEFIs with females (58%) compared to males (28.1%) [3]. Likewise, a study assessing the incidence and risk factors of AEFIs following the first dose of AZ conducted in India found a higher incidence of systemic AEFIs among female healthcare workers, physicians, and those with a history of COVID-19 infection [37]. In addition, according to a Vietnamese study, the percentage of individuals who complained of having symptoms following the AZ vaccine was higher in females than in males [38]. However, Abu-Hammad et al. found that gender was not statistically (*p* > *0.05*) associated with the incidence of AEFI after either the first or second dose of COVID-19 vaccination (Sinopharm, AZ and PZ). This could be due to the small sample of participants (*n* = 409) who took part of their study [39]. Furthermore, relationship between vaccine response and gender differences have been described in the literature. In fact, women have been shown to have stronger immune response to vaccines and effective innate and adaptive immune responses [40]. This could justify why women are more prone to experience AEFIs. Nevertheless, thorough investigations are needed to understand the disparity in the incidence of AEFIs between males and females.

The mean age of vaccine recipients who reported AEFIs in this study was 42.6 ± 17.3 years with a higher incidence among vaccine recipients aging between 18 and 44 years. Similarly, Tran et al. found a decrease of AEFI reporting following vaccination with AZ in Vietnam with the increase of age; the lowest incidence was in people aging more than 60 [38]. Likewise, in Afghanistan, Azimi et al. reported a higher prevalence of AZ adverse reactions in participants aging 40 years or less (94.3%) as compared to those aging more than 40 years (92.5%) [41]. Studies have shown that younger age have been associated with stronger immune reactions and a higher ability to establish an effective response to vaccination which could be the reason behind our findings [4].

In terms of AEFIs reporting rate, it is worth noting that in this study AEFIs with COVID-19 vaccines reporting rates per 1000 doses administered for both PZ and AZ vaccines are higher than those of Ontario, Canada. For instance, 4254 cases following the PZ vaccine were reported to the national PV program in Lebanon which translates into a reporting rate of 0.99 per 1000 doses administered as opposed to Ontario, where it has recorded a reporting rate of 0.61 per 1000 doses administered of the same vaccine. The large number of doses administered in Ontario may have led to the lower reporting rate. Indeed, since the deployment of COVID-19 vaccines, 30, 747, 250 doses have been administered in Ontario until February 13th, 2022, whereas only 5,134,093 doses have been administered in Lebanon until February 19th, 2022 [42, 43].

This analysis revealed that the most common adverse events for both the PZ and AZ COVID-19 vaccines were injection site pain, fatigue, general body pain, headache, and fever. These findings were consistent with the study by Beatty et al. that analyzed adverse events following COVID-19 vaccine [44]. In our study, AEFIs were more frequently reported following the AZ vaccine. This observation agreed with the study by Al Khames Aga et al. done on Iraqi and Jordanian participants which revealed a higher incidence of AEFIs with the AZ vaccine compared to the PZ and Sinopharm vaccines [45]. It is worth noting that gastrointestinal (GI) symptoms, mainly nausea (AZ: 22.9% vs PZ: 12.4%), abdominal pain (AZ: 10.5% vs PZ: 6.4%), diarrhea (AZ: 8.7% vs 6.2%), and vomiting (AZ: 7.6% vs PZ: 4.3%), were significantly associated with the AZ vaccine as compared to the PZ vaccine. In accordance with these findings, a clear association between GI symptoms (diarrhea, nausea, and vomiting) and the AZ vaccine was found in a Jordanian study by Abu-Hammad et al. [39].

Following the PZ vaccine, a study by El-Shitany et al. showed that injection site pain, headache, fatigue, and fever were the main symptoms among Saudi residents, which were in accordance with findings of the present study [3]. In addition, the results were consistent with the adverse events reported by healthcare workers in Jordan [39]. Furthermore, a study by Hause et al. revealed that the most commonly reported reactions after the second dose of the PZ vaccine were also in line with our results [46]. Adding to this, an original article by Polack et al. assessing the safety of PZ mRNA COVID-19 vaccine revealed that fatigue and headache were the most common AEFIs reported after the second dose [47]. Moreover, in agreement with our results, a study by Anderson et al. showed that adverse events mostly reported after the Moderna vaccine, an mRNA COVID-19 vaccine, such as the PZ vaccine, were tiredness, chills, headache, muscle pain, and injection-site pain. Their study revealed that these AEFIs were dose-dependent and were predominantly reported following the second dose which is also in line with our findings [48].

As for local AEFIs in the study by Polack et al., a remarkably smaller percentage reported redness and swelling at the injection site which is similar to our findings. Nevertheless, they have found that injection site pain was more common following the first dose as compared to the second dose [47]. In contrast to our study, pain at the injection site was more frequently reported after the second dose. Concerning the dose number, analogous results were found in a study by Andrzejczak et al., where participants who received their second dose reported more adverse events than those who received their first dose [49]. This could be explained by the greater immunogenicity following sensitization to the viral antigen after the first dose. Indeed, several studies have concluded that more adverse reactions, especially systemic AEFIs, were found following the second dose of PZ vaccine as opposed to the first dose [50].

On the other hand, concerning the AEFIs with the AZ vaccine, our findings were aligned with results from Afghanistan, which reported muscle pain and fatigue as the most common systemic adverse effects, and injection site pain followed by injection site swelling as most common local adverse event [41]. Furthermore, healthcare workers in Nepal reported similar reactions after receiving their dose of AZ vaccine [51]. Moreover, in comparison with our findings, a randomized controlled trial (RCT) conducted by Voysey et al. in Brazil, South Africa, and the UK studying the safety and efficacy of the AZ vaccine revealed that local injection site reactions, mainly pain and tenderness at the injection site, were most frequently reported following the AZ vaccine [52]. Furthermore, based on a study by Nguyen et al., Vietnamese adults reported similar AEFIs following the administration of the AZ vaccine [38]. One important finding of our study is that injection site pain, general body pain, fatigue, chills, and nausea following the AZ vaccine were significantly associated with the administration of the 1st dose. This finding was in line with a study by Jeon et al. that showed that AEFIs associated with the second dose of AZ vaccine were less common compared to the first dose [4].

As for the serious AEFIs, the finding of low rates is consistent with data from ongoing clinical trials and safety surveillance system [44, 47, 53]. Out of the 68 serious cases reported to the PV program, 4 were diagnosed with Guillain–Barre Syndrome (GBS) after the receipt of the PZ vaccine. The United Kingdom’s Medicine and Healthcare products Regulatory Agency (MHRA) added a warning on GBS to the information leaflet of the AZ Vaccine following several reports from different countries [54]. However, our cases of GBS were seen following the PZ vaccine. Many reports demonstrated an immunological pathophysiology of GBS following vaccination against multiple pathogens, such as influenza vaccine, hepatitis B and A, tetanus, and polio vaccines [55]. Nevertheless, in an interim analysis of surveillance data conducted in the United States, GBS incidence in the 21 days after PZ mRNA vaccination was 1.4 per 100,000 person-years, which was similar to the overall expected background rate. Therefore, results showed that mRNA vaccines do not appear to be associated with GBS [56]. Another serious AEFI reported was Vaccine Induced Thrombotic Thrombocytopenia (VITT) following the first dose of AZ vaccine. After several reports from multiple countries across Europe, the European Medicine Agency (EMA) confirmed the rare risk of thrombocytopenia and unusual blood clots associated with the AZ vaccine [57]. Published estimates of VITT after the first dose of AZ across countries with moderate to high data quality ranges between 1 per 26,500 to 1 per 127,300 [58]. In Lebanon, the risk of VITT with AZ vaccine as of February 19th, 2022, has been estimated to be approximately 1 per 717,370 doses, which is much lower than other countries. As for thrombotic events, a study conducted by Tobaiqy et al. regarding venous thrombosis, thrombocytopenia and ischemic stroke related to COVID-19 vaccines showed that 58.1% were related to the AZ vaccine and 32% to the PZ vaccine. In contrast, in Lebanon, 6 thrombotic adverse events were reported in this current study, with 66% temporally associated with the PZ vaccine and 33% with the AZ vaccine [59]. Myocarditis on the other hand, has been reported as a rare adverse event following mRNA vaccines against COVID-19. After the start of mass immunization campaigns against COVID-19, two cases of myocarditis were reported to the LNPVP after receiving the PZ vaccine, an mRNA vaccine. One potential mechanism suggested by Heymans and Cooper is that the immune system may identify mRNA vaccines as antigen, leading to the activation of immunological pathways and pro-inflammatory cascades in the heart. Another plausible mechanism is the molecular mimicry between cardiac self-antigens and the spike protein of the Severe Acute Respiratory Syndrome Coronavirus 2 (SARS-CoV-2) [60]. In a cohort-study based on Denmark, myocarditis had an absolute rate of 1.7 per 100,000 vaccinated persons within 28 days of any mRNA COVID-19 vaccine [61]. In Canada, 1886 cases of myocarditis or pericarditis have been reported to the Public Health of Canada as of March 4, 2022; 1192 of the reported cases were seen after the PZ which is in accordance with our findings [62]. In addition, several published case reports have suggested a possible relationship between myocarditis and mRNA COVID-19 vaccines [63].

Moreover, a retrospective cohort study by Wong et al. revealed that there is a higher risk of myocarditis in men aged between 18 and 25 years following the second dose of mRNA vaccines [64]. In addition, a study by Witberg et al. revealed that male vaccine recipients aging between 16 and 29 years had the highest incidence of myocarditis which have reached 10.7 cases per 100,000 [65]. The demographic characteristics were in line with the myocarditis cases reported to the LNPVP, since they occurred in a 24- and 25-year-old male patients.

Furthermore, a review article by Das et al. described 29 cases of myocarditis reported in the literature out of which only 10% were after the 1st dose [66]. In the Lebanese context, the two cases of myocarditis were reported following the PZ, the first case was after the 1st dose, and the second case was after the second dose. In terms of clinical presentation, both cases of myocarditis presented with gastrointestinal symptoms unlike reports published in the literature, whereby chest pain was the most common presenting symptom [16, 63, 67].

The overall similarity between the results of this study and results from other countries indicates the safety profiles of vaccines used in Lebanon. Public vaccine confidence can be boosted by greater dissemination of knowledge about the overall low incidence of serious cases.

Several limitations of this retrospective study should be addressed. First, underreporting is a main constraint of passive surveillance systems worldwide [68]. Nevertheless, it is important to note that reporting by healthcare professionals has increased from 6 to 13.6% within 6 months according to the monthly report published by the PV department titled “Adverse Events Following Immunization for COVID-19 vaccines in Lebanon” [42]. This increase was mainly due to on-site awareness about the importance of reporting and capacity buildings completed by the PV team to the concerned parties. Emphasis on the fact that AEFIs should be expected and may occur independently of the immunization staff in action was essential to be clarified, where the fear of being penalized may be the major reason of underreporting [69]. In addition, most AEFIs were self-reported which may be subject to bias, since consumers may not be able to accurately assess themselves. However, our study is unique in providing insight on the common and serious AEFIs with COVID-19 vaccines in Lebanon which plays an essential role in decreasing vaccine skepticism among the public.

## Conclusion

Adverse events reported after COVID-19 vaccination in Lebanon were similar to reports found worldwide. Our findings help boost the public’s confidence in the healthcare system, and address concerns raised about the serious adverse events following COVID-19 vaccines. Although the evidence presented in this study indicated that COVID-19 vaccines have acceptable safety profiles, it is still too early to say that they are entirely safe. Additional and larger studies are required to monitor long-term adverse reactions of these vaccines. Nevertheless, several steps can be taken to strengthen the COVID-19 vaccine safety surveillance and encourage immunization among the general population. These would include an active vaccine safety surveillance system, appropriate awareness programs, and the implementation of risk minimization actions.

## Data Availability

Not applicable.
